# Role of *IL-17* Variants in Preeclampsia in Chinese Han Women

**DOI:** 10.1371/journal.pone.0140118

**Published:** 2015-10-09

**Authors:** Haiyan Wang, Mingzhen Guo, Fenghua Liu, Jingli Wang, Zheng Zhou, Jing Ji, Yuanhua Ye, Weiqing Song, Shiguo Liu, Bo Sun

**Affiliations:** 1 Department of Blood Transfusion, the Affiliated Hospital of Qingdao University, Qingdao, China; 2 Department of Blood Transfusion, the First Affiliated Hospital of Harbin Medical University, Harbin, China; 3 Prenatal Diagnosis Center, the Affiliated Hospital of Qingdao University, Qingdao, China; 4 Department of Pathogenic Microorganisms, the Medical College of Qingdao University, Qingdao, China; 5 Department of Clinical Laboratory, Qingdao Municipal Hospital (Group), Qingdao, China; St Francis Hospital, UNITED STATES

## Abstract

Previous studies have suggested an important role for IL-17, mainly secreted by Th17 cells, in the development of systemic inflammation in preeclampsia (PE). This study therefore investigated the association between genetic variants in *IL-17A*, *IL-17F*, and *IL-17RA* and susceptibility to PE in Chinese Han women. We recruited 1,031 PE patients and 1,298 controls of later pregnant women, and used TaqMan allelic discrimination real-time PCR to genotype the polymorphisms of IL17A rs2275913, IL-17F rs763780, and IL-17RA rs4819554. No significant differences in genotypic or allelic frequencies were found at all three polymorphic sites between PE patients and controls (rs2275913: genotype χ^2^ = 0.218, *p* = 0.897 and allele χ^2^ = 0.157, *p* = 0.692, OR = 1.024, 95%CI 0.911–1.152; rs763780: genotype χ^2^ = 1.948, *p* = 0.377 and allele χ^2^ = 1.242, *p* = 0.265, OR = 0.897, 95%CI 0.741–1.086; rs4819554: genotype χ^2^ = 0.633, *p* = 0.729 and allele χ^2^ = 0.115, *p* = 0.735, OR = 1.020, 95%CI 0.908–1.146). There were also no significant differences in genetic distributions between mild/severe PE or early/late-onset PE and control subgroups. Our data indicate that the genetic variants of rs2275913 in *IL-17A*, rs763780 in *IL-17F*, and rs4819554 in *IL-17RA* may not play a role in the pathogenesis of PE in Chinese Han women. However, these findings should be confirmed in other ethnic populations.

## Introduction

Preeclampsia (PE) affects about 3–7% of pregnancies and is characterized by the new onset of hypertension with proteinuria after the 20^th^ week of gestation [1]. It can seriously threaten the health of both the mother and the fetus and is a leading cause of maternal and perinatal morbidity and mortality worldwide [2]. It involves an excessive inflammatory response of the matrix to pregnancy [3] caused by an immune system imbalance [4,5]. Th17 cells, a subset of CD4+ T helper cells, are characterized by their secretion of IL-17. This can combine with Th17 receptors and promote neutrophils to recruit and further induce the production of many other inflammatory cytokines such as tumor necrosis factor (TNF)-α, IL-1, IL-6, and IL-8 [6], all of which participate in the development of PE. Gergely et al. previously demonstrated that Th17 cells were elevated in PE women compared with healthy pregnant women [7], and this was confirmed by Cornelius and his colleagues [8]. Because an infusion of IL-17 inhibitor into PE rats significantly decreased blood pressure and placental oxidative stress [9], we hypothesized that *IL-17* might play a pivotal role in the pathogenesis of PE.

The IL-17 cytokine family consists of six members in mammals, IL-17A–F, which are encoded by *IL-17A*–*F* respectively. IL-17A and IL-17F, located adjacent to each other on chromosome 6p12 with an approximately 50% sequence identity, are two important pro-inflammatory cytokines that are crucial in the development of many chronic inflammatory diseases [10]. The IL-17 receptor (IL-17R) family comprises five receptor subunits, IL-17RA–E, which are encoded by *IL-17RA*–*E* respectively. Located on chromosome 22q11.1, IL-17RA is the largest member by far and can combine with many cytokines including IL-17A and IL-17F, and then induce the expression of other inflammatory factors [11]. The single nucleotide polymorphisms (SNPs) IL-17A rs2275913 (-197G/A) and IL-17RA rs4819554 (-809A/G) are located in promoter region [12,13] and IL-17F rs763780 (7488T/C) is within exon 3, all three SNPs can influence the expression of *IL-17* and *IL-17R*. Liu XK and his colleagues [12] identified that the region between -232 and -159 of *IL-17A* has an inducible promoter activity. Kawaguchi [14] et al. have shown that the IL-17F rs763780 can cause a His-to-Arg substitution at amino acid 161 (H161R), thus influence the risk of asthma and is a natural IL-17F antagonist in the known polymorphisms of *IL-17*. In addition, the rs2275913, rs763780 and rs4819554 are all tag SNPs and most frequently studied among the enormous genes of IL-17A, IL-17F and IL-17RA, and previous studies indicated that these three SNPs are associated with diseases such as recurrent pregnancy loss (RPL), rheumatoid arthritis (RA), inflammatory bowel disease, and kidney disorders [13,15–17]. The present study therefore investigated the association between SNPs rs2275913, rs763780, and rs4819554 and susceptibility to PE in Chinese Han women.

## Materials and Methods

### Subjects

We enrolled 1,031 PE patients and 1,298 normal later pregnant women admitted to the Affiliated Hospital of Qingdao University, Binzhou Medical University Hospital, Yantai Yuhuangding Hospital, Yantaishan Hospital, Linyi People’s Hospital, Liaocheng People’s Hospital, and the Maternal and Child Health Care of Zaozhuang between January 2012 and November 2014. Demographic and clinical characteristics including maternal age, gestational week, blood pressure, pregnancy and delivery history, clinical symptoms, and results of laboratory examinations were collected in a clinical database. All recruited subjects were Chinese Han women, and controls were age-matched to the PE patients within one year. The present study was approved by the ethics committee of the Affiliated Hospital of Qingdao University and all PE patients and normal controls provided their written informed consent.

The diagnosis of PE was based on the onset of hypertension (≥140/90mmHg) with proteinuria (≥0.3g/24h, or ≥1+ by dipstick) after 20 weeks of gestation in a woman with previously normal blood pressure, and could be accompanied by symptoms such as upper abdominal discomfort, headache, and blurred vision, according to previously published criteria [18]. Inclusion criteria for controls were as follows:1) age≥26 years,2) gestational age≥30 weeks,3) no clinical history of PE, chronic hypertension, heart disease, kidney disorders, diabetes mellitus, hepatic diseases, transfusion, or immunotherapy, and 4)without obstetric complications such as premature membrane rupture, placenta previa, threatened abortion, artificial insemination, twin or multiple pregnancy, and macrosomia in the present gestation.

### Genetic studies

Genomic DNA was extracted from 300 μl peripheral venous blood using a Qiagen DNA extraction kit (Qiagen, Hilden, Germany). Genotyping for polymorphisms of IL17A rs2275913, IL-17F rs763780, and IL-17RA rs4819554 was conducted by the TaqMan allelic discrimination real-time PCR. Taqman probes and primers were synthesized by Applied Biosystems of Life Technologies (New York, USA). The rs275913 primers were 5′-TGCCCTTCCCATTTTCCTTCAGAAG-3′ (forward) and 5′-AGAGATTCTTCTATGACCTCATTGG-3′ (reverse); rs763780 primers were 5′-GTGGATATGCACCTCTTACTGCACA-3′ (forward) and 5′-GGTGGATGACAGGGGTGACGCAGGT-3′ (reverse); and rs4819554 primers were 5′-GGGAAGTAACGACTCTCTTAGGTGC-3′ (forward) and 5′-GCTGGGACACAGTCTCACAGACCAG-3′ (reverse). PCR was conducted in a 25-μl reaction system containing 1.25 μl 20×SNP Genotyping Assay, 12.5 μl 2×PCR Master Mix, and 11.25 μl DNA and DNase-free water. Amplifications were carried out in aC1000™ thermal cycler and CFX96™ real-time system (Bio-Rad, California, USA) under the following conditions: 95°C for 3 min, followed by 45 cycles of 95°C for 15 s and 60°C for 1 min. For each cycle, the fluorescent signals from VIC/FAM-labeled probes were detected. The discrimination of genotypes was conducted using Bio-Rad CFX manager 3.0 software.

### Statistical analysis

All analyses were performed using statistical software package SPSS 21.0(SPSS Inc., Chicago, IL, USA). Student’s *t*-test was used to compare differences in demographic and clinical characteristics between cases and controls. The chi-square test examined the Hardy—Weinberg equilibrium (HWE) in the control group to ensure that it was representative. Differences in genotypic and allelic frequencies between cases and controls were compared by Pearson’s chi-square test (Fisher’s exact test was used when expected values were below 5). We used odds ratios (ORs) and 95% confidence intervals (CIs) to show the relative risk degree. Statistical significance was set at *p*<0.05. While the power analysis was performed with the program Power and Sample Size Calculations (PS, Version 3.1.2), considering an alpha of 0.05.

## Results

### Demographic and Clinical Characteristics

Table 1 shows a comparison of demographic and clinical characteristics between cases and controls. Both groups were age-matched with mean ± SD ages of 30.00±5.79 years for cases, and 30.34±4.11years for controls (*p* = 0.107). No significant differences in gravidity, number of abortions, and age of menarche were observed between cases and controls (*p*>0.05). However, PE patients were admitted and delivered at a significantly earlier number of gestational weeks (*p*<0.001), and had significantly lower fetal birth weights (*p*<0.001), higher blood pressure (*p*<0.001), and elevated levels of white blood cells (*p*<0.001) and neutrophils (*p* = 0.001) compared with controls.

**Table 1:** The demographic and clinical characteristics of PE and control groups.

Characteristics	PE(N = 1031)	Control(N = 1298)	t	*p*-value
Maternal age(years)	30.00±5.79	30.34±4.11	-1.612	0.107
Times of gravidity	2.22±1.28	2.23±1.19	-0.161	0.872
Number of abortion	0.65±0.95	0.65±0.87	0.035	0.972
Age of menarche(years)	13.99±1.30	14.10±1.30	-1.869	0.062
Gestational age at admission(weeks)	35.10±5.11	38.91±2.70	-21.086	<0.001
Gestational age at delivery(weeks)	35.90±4.83	39.11±3.28	-17.285	<0.001
Fetal birth weight (kg)	2.61±0.92	3.40±0.38	-24.479	<0.001
Systolic blood pressure(mmHg)	158.74±18.84	113.69±10.70	68.160	<0.001
Diastolic blood pressure(mmHg)	103.74±13.79	73.41±7.85	62.681	<0.001
White blood cell(×10^9^/L)	9.66±3.09	9.02±2.62	5.278	<0.001
Neutrophil(×10^9^/L)	7.45±4.77	6.86±3.00	3.421	0.001

### Analysis of Genotypic and Allelic Frequencies

The controls in our study were in accordance with HWE (IL-17A rs2275913, χ^2^ = 2.564, *p* = 0.109; IL-17F rs763780, χ^2^ = 0.337, *p* = 0.561; IL-17RA rs4819554, χ^2^ = 0.043, *p* = 0.836).Table 2 shows the genetic distributions of rs2275913, rs763780, and rs4819554 between cases and controls. No significant differences were observed at the three polymorphic sites between the two groups in terms of genotypic distributions (rs2275913, χ^2^ = 0.218, *p* = 0.897; rs763780, χ^2^ = 1.948, *p* = 0.377; rs4819554, χ^2^ = 0.633, *p* = 0.729), nor for allelic frequencies (rs2275913, χ^2^ = 0.157, *p* = 0.692, OR = 1.024, 95%CI 0.911–1.152; rs763780, χ^2^ = 1.242, *p* = 0.265, OR = 0.897, 95%CI 0.741–1.086; rs4819554, χ^2^ = 0.115, *p* = 0.735, OR = 1.020, 95%CI 0.908–1.146).

**Table 2:** The comparison of genotypic and allelic frequencies between PE and control groups.

Group	N	rs2275913					rs763780					rs4819554				
		AA	AG	GG	A	G	CC	CT	TT	C	T	AA	AG	GG	A	G
PE	1031	192	473	366	857	1205	8	185	838	201	1861	319	520	192	1158	904
Control	1298	232	600	466	1064	1532	17	245	1036	279	2317	404	637	257	1445	1151
χ^2^		0.218			0.157		1.948			1.242		0.633			0.115	
*p*-value		0.897			0.692		0.377			0.265		0.729			0.735	
OR					1.024					0.897					1.020	
95%CI				0.911–1.152					0.741–1.086					0.908–1.146		

To further investigate the association between *IL-17* variants and PE, we divided PE patients into mild and severe PE groups according to guidelines from the American College of Obstetricians and Gynecologists [19]. We then compared the genotypic and allelic frequencies of rs2275913, rs763780, and rs4819554 in mild or severe PE with those in control groups. Table 3 shows that no significant differences between mild/severe PE patient and controls’ genetic distributions were found at any of the three polymorphic sites (mild PE vs. control: rs2275913, χ^2^ = 2.346, *p* = 0.309 by genotype, χ^2^ = 2.420, *p* = 0.120 by allele; rs763780, *p* = 0.838 by genotype, χ^2^ = 0.490, *p* = 0.484 by allele; rs4819554, χ^2^ = 1.046, *p* = 0.593 by genotype, χ^2^ = 0.001, *p* = 0.978 by allele; severe PE vs. control: rs2275913, χ^2^ = 0.093, *p* = 0.954 by genotype, χ^2^ = 0.046, *p* = 0.830 by allele, rs763780, χ^2^ = 1.785, *p* = 0.410 by genotype, χ^2^ = 1.009, *p* = 0.315 by allele; rs4819554, χ^2^ = 0.312, *p* = 0.855 by genotype, χ^2^ = 0.151, *p* = 0.697 by allele).

**Table 3:** The comparison of genetic distributions between mild/severe PE and control groups.

Group	N	rs2275913					rs763780					rs4819554				
		AA	AG	GG	A	G	CC	CT	TT	C	T	AA	AG	GG	A	G
Mild PE	218	47	102	69	196	240	2	38	178	42	394	64	115	39	243	193
Control	1298	232	600	466	1064	1532	17	245	1036	279	2317	404	637	257	1445	1151
χ^2^		2.346			2.420					0.490		1.046			0.001	
*p*-value		0.309			0.120		0.838^a^			0.484		0.593			0.978	
OR					1.176					0.885					1.003	
95%CI				0.959–1.442					0.629–1.246					0.818–1.230		
Severe PE	813	145	371	297	661	965	6	147	660	159	1467	255	405	153	915	711
Control	1298	232	600	466	1064	1532	17	245	1036	279	2317	404	637	257	1445	1151
χ^2^		0.093			0.046		1.785			1.009		0.312			0.151	
*p*-value		0.954			0.830		0.410			0.315		0.855			0.697	
OR					0.986					0.900					1.025	
95%CI				0.869–1.119					0.733–1.105					0.905–1.161		

^a^: *p*-value of Fisher’s exact test

Early-onset PE patients were those diagnosed before the 34^th^ week of gestation, and are known to be more severely affected than those with later-onset PE [20].Table 4 shows that there were no significant differences in the genetic distributions ofrs2275913, rs763780, and rs4819554 between early/late-onset PE and control groups (early-onset PE vs. control: rs2275913, χ^2^ = 0.268, *p* = 0.861 by genotype, χ^2^ = 0.047, *p* = 0.828 by allele; rs763780, *p* = 0.750 by genotype, χ^2^ = 0.023, *p* = 0.800 by allele; rs4819554, χ^2^ = 1.699, *p* = 0.428 by genotype, χ^2^ = 1.562, *p* = 0.211 by allele; late-onset PE vs. control: rs2275913, χ^2^ = 0.330, *p* = 0.848 by genotype, χ^2^ = 0.155, *p* = 0.694 by allele; rs763780, χ^2^ = 3.645, *p* = 0.162 by genotype, χ^2^ = 1.291, *p* = 0.256 by allele; rs4819554, χ^2^ = 2.037, *p* = 0.361 by genotype, χ^2^ = 1.245, *p* = 0.265 by allele).

**Table 4:** The comparison of genetic distributions between early/late-onset PE and control groups.

Group	N	rs2275913					rs763780					rs4819554				
		AA	AG	GG	A	G	CC	CT	TT	C	T	AA	AG	GG	A	G
Early-onset PE	299	57	134	108	248	350	5	53	241	63	535	82	152	65	316	282
Control	1298	232	600	466	1064	1532	17	245	1036	279	2317	404	637	257	1445	1151
χ^2^		0.268			0.047					0.023		1.699			1.562	
*p*-value		0.861			0.828		0.750^a^			0.800		0.428			0.211	
OR					1.020					0.978					0.893	
95%CI				0.852–1.222					0.732–1.306					0.747–1.067		
Late-onset PE	693	131	315	247	577	809	3	127	563	133	1253	223	351	119	797	589
Control	1298	232	600	466	1064	1532	17	245	1036	279	2317	404	637	257	1445	1151
χ^2^		0.330			0.155		3.645			1.291		2.037			1.245	
*p*-value		0.848			0.694		0.162			0.256		0.361			0.265	
OR					1.027					0.882					1.078	
95%CI				0.900–1.172					0.709–1.096					0.945–1.230		

^a^: *p*-value of Fisher’s exact test

## Discussion

PE is one of the most common and severe pregnancy-specific complications. Although it is accepted that placental ischemia and hypoxia, oxidative stress, inflammatory responses, an immune imbalance, and hereditary factors are associated with PE, the precise etiology and pathogenesis of the disease have remained unclear. However, it is becoming increasingly apparent that PE reflects an excessive maternal inflammatory response, with a Th1/Th2 immune imbalance [3–5]. In the present study, we found that PE patients have higher levels of white blood cells and neutrophils compared with controls, supporting the fact that inflammation participates in disease pathophysiology.

Normal pregnancy is considered to be a predominantly Th2 immunological state, which favors an immune-tolerant environment for the prevention of fetal rejection [21]. By contrast, a PE pregnancy is characterized as a maternal pro-inflammatory state with a Th1 predominance and increased plasma levels of pro-inflammatory cytokines, and occurs mainly during the second and third trimesters of pregnancy [22,23]. However, it has been shown that the Th1/Th2 paradigm only partially explains the functional and molecular changes observed during normal or pathological pregnancies, thus the Th1-Th17/Th2-Treg paradigm has been proposed instead [24,25]. Th17 cells, a relatively novel CD4+ lymphocyte subpopulation, are characterized by the secretion ofIL-17which interacts with inflammatory factors to amplify the small vascular inflammatory reactions of the placenta, damage vascular endothelial cells, increase the permeability of blood vessels, and release large numbers of oxygen free radicals. Together, these constitute the pathological basis of the clinical manifestations of PE, including hypertension, vascular spasms, edema, and proteinuria [6,9,25]. Cornelius [9] et al. infused IL-17 soluble receptor C(an IL-17 inhibitor) into rats model of PE, and found that it could significantly decrease blood pressure and blunt Th17 cells and placenta oxidative stress effect. Therefore, we suppose IL-17 might play a significant role in the pathogenesis of PE.

PE is a complex polygenetic hereditary disease, associated with both genetic and environmental factors [26]. Previous studies have shown that cytokine genes such as *VEGF* [27,28], *NLRP1 [29]*, *TNF-α* [30], and *IL-1* [31,32] are associated with PE risk. For instance, Mohammad et al. observed significant differences in the *TNF-α* genotype at position−238 between PE and control groups, and concluded that the A allele may carry an increased risk for developing PE [30]. Moreover, we previously showed that IL-1A rs17561 [31] and IL-1β−31C/T and −511T/C polymorphisms [32] were associated with PE in the Chinese Han population. Additionally, IL-17 can induce the production of cytokines such as TNF-α and IL-1, both of which play important roles in the pathophysiology of PE. Thus, our present study investigated the association between *IL-17*variants and PE in Chinese Han women.

Our study of 1,031 PE patients and 1,298 age-matched controls found no significant differences in genotypic and allelic frequencies of polymorphic sites IL-17A rs2275913, IL-17F rs763780, and IL-17RA rs4819554 between PE and control groups. To further understand the relationship between *IL-17* and PE, we divided the PE patients into mild/severe and early/late-onset groups, but again found no significant differences in genetic distributions at all three polymorphic sites. These data suggest that the polymorphisms of rs2275913 in *IL-17A*, rs763780 in *IL-17F*, and rs4819554 in *IL-17RA*donot play a pivotal role in the pathophysiology of PE, at least in Chinese Han women, which is in accordance with the results of Anvari F and his colleagues [33]. And the post-hoc power calculations of rs2275913, rs763780 and rs4819554 were 5.9%, 12.2% and 5.6% respectively, which indicates that our results are credible given the sufficient sample size of our study. However, previous studies have investigated the association between the three polymorphic sites and risk of other diseases. For instance, Nordang et al. demonstrated that rs2275913 is weakly associated with rheumatoid arthritis in a Norwegian population [15], Najafi et al. indicated that rs763780 might be associated with a high risk of RPL in Iranian women [13], while Kim [34] et al. proposed that rs4819554 greatly affected the risk of end-stage renal disease development.

Our negative results could be explained by many reasons. Because PE is a complex polygenetic hereditary disease, it is conceivable that one or even several genetic defects might not affect gene expression and environmental factors such as diet, obesity, stress, and smoking may instead influence the development of PE. Moreover, regional and racial differences are likely to affect the results. One limitation of our study was that most subjects were from Shandong province. Additionally, we only investigated three SNPs of *IL-17* and did not analyze the interaction with environmental risk factors and comorbidity. Thus, larger-scale functional and genetic studies investigating different genes in patients from multiple regions are necessary to discover new PE susceptibility genes and gain further insights into its pathogenesis.
